# Germline Genetic Variation in *ETV6* and Risk of Childhood Acute Lymphoblastic Leukemia: a Systematic Genetic Study

**DOI:** 10.1016/S1470-2045(15)00369-1

**Published:** 2015-10-28

**Authors:** Takaya Moriyama, Monika L. Metzger, Gang Wu, Rina Nishii, Maoxiang Qian, Meenakshi Devidas, Wenjian Yang, Cheng Cheng, Cao, Quinn Emily, Susana Raimondi, Julie M. Gastier-Foster, Elizabeth Raetz, Eric Larsen, Paul L. Martin, W. Paul Bowman, Naomi Winick, Yoshihiro Komada, Shuoguo Wang, Michael Edmonson, Heng Xu, Elaine Mardis, Robert Fulton, Pui, Charles Mullighan, William E. Evans, Jinghui Zhang, Stephen P. Hunger, Mary V. Relling, Kim E. Nichols, Mignon L. Loh, Jun J. Yang

**Affiliations:** a Department of Pharmaceutical Sciences, St. Jude Children's Research Hospital, Memphis, Tennessee, USA; b Department of Pediatrics, Mie University Graduate School of Medicine, Mie, Japan; c Department of Oncology, St. Jude Children's Research Hospital, Memphis, Tennessee, USA; d Department of Computational Biology, St. Jude Children's Research Hospital, Memphis, Tennessee, USA; e Department of Pediatrics and Developmental Biology, Tokyo Medical and Dental University Graduate School of Medicine, Tokyo, Japan; f Department of Biostatistics, College of Medicine, Public Health & Health Professions, University of Florida, Gainesville, Florida, USA; g Department of Biostatistics, St. Jude Children's Research Hospital, Memphis, Tennessee, USA; h Department of Pathology, St. Jude Children's Research Hospital, Memphis, Tennessee, USA; i Department of Pathology and Laboratory Medicine, Nationwide Children's Hospital, and Departments of Pathology and Pediatrics, Ohio State University, Columbus, Ohio, USA; j Huntsman Cancer Institute, The University of Utah, Salt Lake City, Utah, USA; k Maine Children's Cancer Program, Scarborough, Maine, USA; l Department of Pediatrics, Duke University, Durham, North Carolina, USA; m Cook Children's Medical Center, Ft. Worth, Texas, USA; n Pediatric Hematology Oncology, University of Texas Southwestern Medical Center, Dallas, Texas, USA; o Department of Laboratory Medicine, National Key Laboratory of Biotherapy/Collaborative Innovation Center of Biotherapy and Cancer Center, West China Hospital, Sichuan University, Chengdu, China; p McDonnell Genome Institute, Washington University School of Medicine, St Louis, Missouri, USA; q Hematological Malignancies Program, Comprehensive Cancer Center, St. Jude Children's Research Hospital, Memphis, Tennessee, USA; r Department of Pediatrics and Center for Childhood Cancer Research, Children's Hospital of Philadelphia and the University of Pennsylvania Perelman School of Medicine, Philadelphia, Pennsylvania, USA; s Department of Pediatrics, Benioff Children's Hospital and the Helen Diller Family Comprehensive Cancer Center, University of California at San Francisco, California, USA

## Abstract

**Background:**

Hereditary predisposition is rarely suspected for childhood acute lymphoblastic leukemia (ALL). Recent studies identified germline *ETV6* variations associated with marked familial clustering of hematologic malignancies, pointing to this gene as a potentially important genetic determinant for ALL susceptibility. The aims of the current study are to comprehensively identify ALL predisposition variants in *ETV6* and to determine the extent to which they contribute to the overall risk of childhood ALL.

**Methods:**

Whole-exome sequencing of an index family with multiple cases of ALL was performed to identify causal variants for ALL predisposition. Targeted sequencing of *ETV6* was done in 4,405 children from the Children's Oncology Group (COG) and St. Jude Children's Research Hospital frontline ALL trials. Patients were included in this study on the basis of their enrollment in these clinical trials and the availability of germline DNA. *ETV6* variant genotypes were compared with non-ALL controls to define ALL-related germline risk variants. *ETV6* variant function was characterized bioinformatically and correlated with clinical and demographic features in 2,021 children with ALL.

**Findings:**

We identified a novel nonsense *ETV6* variant (p.R359X) with a high penetrance of familial ALL. Subsequent targeted sequencing of *ETV6* in 4,405 childhood ALL cases discovered 31 exonic variants (4 nonsense, 21 missense, 1 splice site, and 5 frame shift variants) that are potentially related to ALL risk in 35 cases (0.79%). Fifteen (48%) of the 31 ALL-related *ETV6* variants clustered in the ETS domain and predicted to be highly deleterious. Children with ALL-related *ETV6* variants were significantly older at leukemia diagnosis than others (10.2 years [IQR 5.3-13.8] vs 4.7 years [IQR 3.0-8.7], P=0.017). The hyperdiploid leukemia karyotype was strikingly overrepresented in ALL cases harboring germline *ETV6* risk variants compared to the wildtype group (9 of 14 cases [64.3%] vs 538 of 2,007 cases [26.8%]; P=0.0050).

**Interpretation:**

Our findings indicated germline *ETV6* variations as the basis of a novel genetic syndrome associated with predisposition to childhood ALL.

**Funding:**

This study was supported by the National Institutes of Health and by the American Lebanese Syrian Associated Charities.

## Research in context

### Evidence before this study

As the most common cancer in children, acute lymphoblastic leukemia (ALL) is generally considered as driven by acquired genomic abnormalities and hereditary predisposition is rarely considered in clinical practice. Recent studies identified *ETV6* germline variants associated with rare hereditary thrombocytopenia and a plausible increased susceptibility to hematologic malignancies, including ALL.

### Added value of this study

Combining whole-exome discovery in familial ALLs and targeted sequencing in a large frontline national clinical trials (N=4,405), we comprehensively identified a panel of 31 *ETV6* germline variants that were likely associated with ALL risk. Children with ALL-relate *ETV6* variants had distinct clinical features, suggesting unique leukemia etiology. The substantial proportion of childhood ALL cases carrying *ETV6* germline variants of potential pathologic significance highlights the importance of *ETV6* as a leukemia predisposition gene and also indicate that inherited susceptibility to ALL may be much more substantial than currently believed.

### Implications of all the available evidence

Germline *ETV6* variations are the basis for a novel genetic syndrome associated with strong predisposition to hematologic malignancies (particularly ALL), implying potential benefit of clinical identification and subsequent surveillance of the at risk individuals.

## Introduction

Acute lymphoblastic leukemia (ALL) is the most common cancer in children and a prototype of cancer that can be cured by chemotherapy alone with risk-adapted chemotherapeutic regimens.^1-4^ However, the etiology of this aggressive cancer remains largely unknown. The contribution of environmental factors to the development of ALL is debated, although there is growing evidence in support of the influence of exposure to infectious agents early in life.^5-7^ In a Swedish-Finnish population-based study of ∼4,000 patients with childhood ALL, children with affected siblings had a 2- to 4-time increased risk to develop ALL and this risk increased up to 163-time for monozygotic twins,^8, 9^ pointing to a potential genetic basis of susceptibility to ALL. In fact, common germline genetic polymorphisms affecting genes involved in lymphoid development and tumor suppression (e.g., *ARID5B,*^10, 11^ *IKZF1,*^10, 11^ *CEBPE,*^10^ *GATA3,*^12, 13^ *CDKN2A,*^14, 15^ *BMI1-PIP4K2A,*^16^ *TP63*^17^) have been associated with the risk of developing ALL, although with mostly modest effects. Only a small fraction of ALL cases are thought to be related to congenital genetic disorders (e.g., Down syndrome^18, 19^, Robertsonian translocation^20^), and hereditary predisposition is rarely considered in clinical practice. Recent studies of familial ALL have identified rare germline mutations in *PAX5* and *SH2B3* with high penetrance.^21, 22^ More strikingly, germline *TP53* mutations were found in ∼50% of children with low-hypodiploid ALL, suggesting that this subtype of ALL may be a manifestation of Li Fraumeni syndrome.^23^ Together, these data raise the possibility that the proportion of ALL cases attributable to inherited genetic mutations may be much higher than currently proposed.

*ETV6* is a transcriptional repressor that belongs to the ETS family and is essential for hematopoiesis, particularly thrombopoiesis.^24, 25^ Somatic *ETV6* mutations have been associated with myelodysplastic syndromes and T-cell leukemias.^26, 27^ In childhood ALL, the *ETV6*-*RUNX1* fusion is the most common somatic genetic aberration and is associated with a good outcome with modern therapy.^28^ Germline *ETV6* variations have recently been reported in rare families with hereditary thrombocytopenia and susceptibility to hematologic malignancies (particularly ALL).^29-31^ These highly damaging variants result in loss of functional *ETV6* protein in a dominant negative fashion,^29-31^ although the exact biological mechanism of excessive ALL risk in these subjects remains unclear. These recent observations raise the questions as to whether additional ALL predisposition variants in *ETV6* exist and to what extent they contribute to ALL risk in general.

In the present study, we identified a novel damaging *ETV6* germline variant driving predisposition to familial ALL, described a population-based screen of *ETV6* germline variation in 4,405 children with ALL, and evaluated the potential functions of ALL-related *ETV6* variants and their association with clinical features of ALL.

## Methods

### Subjects and samples

**Figure 1:**
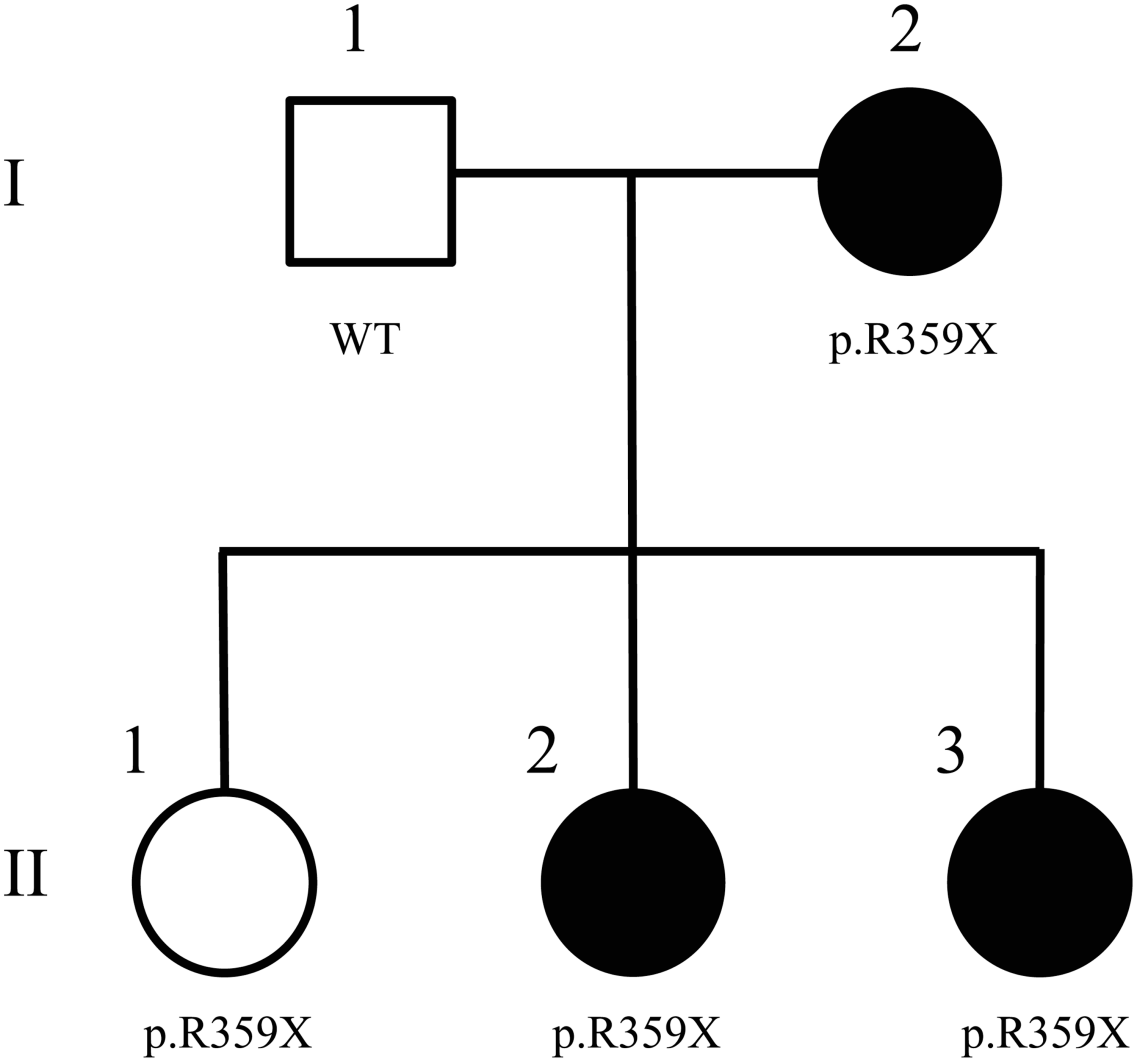
A novel nonsense germline variant in *ETV6* (p.R359X) associated with familial ALL Filled symbols represented individuals with ALL. “WT” and “p.R359X” indicate the wildtype and heterozygous genotype at this *ETV6* variant, respectively.

A family of European ancestry with multiple ALL cases was identified at St. Jude Children's Research Hospital, Memphis USA (Fig. 1). ALL cases were treated on the St. Jude frontline ALL protocols Total Therapy XIIIA^32^, XIIIB^33^ and XV.^34^

The *ETV6* targeted-sequencing cohort comprised 4,405 children with newly-diagnosed ALL (3,807 with B-ALL and 93 with T-ALL) who were treated on the Children's Oncology Group (COG) AALL0232, P9904, P9905, P9906 protocols^35, 36^ and St. Jude Total Therapy XIIIA, XIIIB and XV studies. Individuals in the NHLBI GO Exome Sequencing Project cohort^37^ (ESP, http://evs.gs.washington.edu/EVS/) served as the primary non-ALL control cohort because the prevalence of childhood ALL is extremely low in the general population. In addition, we utilized the Broad Institute Exome Aggregation Consortium cohort [ExAc, http://exac.broadinstitute.org/] as a secondary non-ALL cohort, as it has a considerably larger sample size with greater diversity in ancestry.

Germline DNA was extracted from bone marrow samples or peripheral blood of children with ALL obtained during remission. Most of the ALL cases had been previously genotyped with genome-wide SNP arrays and genetic ancestry (European, African, Native American, and Asian) was estimated using STRUCTURE^38^ on the basis of genotypes at 30,000 randomly selected SNPs.^12, 39, 40^ This study was approved by the appropriate institutional review boards and informed consent was obtained from parents, guardians, or patients, as appropriate.

### Exome sequencing and analyses of the pedigree with familial ALL

Germline genomic DNA was subjected to exome capture (62 Mb) with the Illumina TruSeq kit. Exome sequencing (pair-end 101 bp) was performed on the Illumina HiSeq 2500 platform with a coverage of 10× for > 84% ∼ 94% of target regions. Sequencing reads were mapped using the Burrow-Wheller Aligner and variant calls in the gene coding regions were made by bambino^41^ (with parameters: -min-quality 20, -min-flanking-quality 20, -min-alt-allele-count 3, -min-minor-frequency 0, -broad-min-quality 10, -mmf-max-hq-mismatches 4, -mmf-max-hq-mismatches-xt-u 10, -mmf-min-quality 15, -mmf-max-any-mismatches 6, -unique-filter-coverage 2). Sequencing results were also analyzed using the GATK pipeline (version 3.1)^42^ for calling single nucleotide variants and insertions and deletions. All germline variants were subjected to rigorous quality control including checking for paralogs, repeats, and low variant allele frequency. Using an in-house integrated variant prioritization algorithm, variants were classified into multiple tiers of differing importance on the basis of allele function prediction (e.g., polyphen2, SIFT, mutation assessor, protein truncating, etc), gene function (e.g., known cancer genes in COSMIC^43^, ACMG disease predisposition genes^44^), prior biological evidence for pathologic effects (e.g., HGMD^45^, ClinVar^46^), etc. Variants were evaluated for co-segregation with ALL based on an autosomal dominant mode of inheritance: we hypothesized that the risk variant should be present in subjects with ALL (I-2, II-2, and II-3) and absent in I-1; Because it is not uncommon for ALL to be diagnosed in older children and adolescents, we reason that there is a distinct possibility that II-1 would eventually develop ALL. As a result, we did not require risk variant to be absent in II-1 (appendix p 7). Variants were then filtered based on frequency in non-ALL controls and known gene function in cancer and/or hematopoiesis to identify the final candidates related to cancer risk (appendix p 2, 7).

### Targeted *ETV6* sequencing and function prediction

Illumina dual-indexed libraries were created from the germline DNA of 4,405 children with ALL, and pooled in sets of 96 prior to hybridization with customized Roche NimbleGene SeqCap EZ probes to capture the *ETV6* genomic region. Quantitative PCR was used to determine the appropriate capture product titer necessary to efficiently populate an Illumina HiSeq 2000 flowcell for paired-end 2×101 bp sequencing. A coverage of >20× depth was achieved across >90% of the targeted regions for nearly all samples. Sequence reads in FASTQ format were mapped and aligned using the Burrow-Wheller Aligner, and genetic variants were called using the GATK pipeline (version 3.1), as previously described.^14, 42^

Potential damaging effects of *ETV6* variants were predicted using the combined annotation dependent depletion (CADD, v1.0)^47^ and each variant was assigned a CADD phred-like score. A CADD phred-like score of 10, 20, or 30 represents the top 10%, 1%, and 0.1% of the most deleterious variants, respectively.

Each *ETV6* variant identified in the ALL cohort was curated manually and classified as “ALL-related” or “common” to indicate its potential role in predisposition to ALL, on the basis of variant frequency in ancestry-matched ALL vs. non-ALL cohorts (appendix p 8). For example, variants that were observed only in the ALL cohort were most likely to be related to ALL risk, whereas variants common in non-ALL cohorts were less likely to confer predisposition to this cancer.

### Statistical analyses

The St. Jude Total Therapy protocols and the COG P9900 studies (P9904, P9905, and P9906 protocols) are frontline clinical trials for newly-diagnosed ALL across diverse risk groups and demographics.^32-35^ As such, they were used in population-based analyses of the association of *ETV6* genotype with ALL clinical features. Patients were first classified as “with ALL-related *ETV6* variants” or “without”. The association of germline *ETV6* status with categorical clinical features (tumor translocation [*ETV6-RUNX1, E2A-PBX1, BCR-ABL1, MLL* rearrangements], hyperdiploidy [DNA index ≥1.16], and leukocyte count [< or ≥ 50×10^9^/l]) was determined by using Fisher's exact test. Age at diagnosis (as a continuous variable) was compared between two *ETV6* groups by using the non-parametric Wilcoxon rank sum test because of age did not follow normal distribution. Association of *ETV6* status and genetic ancestry was tested using a multivariate Firth logistic regression model with European, African, and Asian ancestry as covariates. Relapse was treated as a time-dependent variable and its relationship with the status of germline *ETV6* risk variant was evaluated by using the Fine and Gray hazard regression model^48^. Early treatment response was measured as minimal residual disease at the end of remission induction therapy and positivity was defined as ≥ 0.01%^33-35^, and its association with germline *ETV6* status was evaluated by using the Fisher's exact test. Over-representation of ALL-related *ETV6* variants in the ETS domain was evaluated by comparing the observed frequency with what would be expected if variants were randomly distributed in this gene, and the statistical significance was determined by using Fisher's exact test. R (version 3.1) was used for all statistical analyses unless otherwise indicated.

### Role of the funding source

The funding source was not directly involved in the design of the study, the collection, analysis, and interpretation of the data, the writing of the manuscript, or the decision to submit the manuscript. TM, GW, MQ, WY, CC, XC, SR, SW, ME, HW, EM, RF, CM, JZ, MVR, JJY had access to the raw data. The corresponding author had full access to all of the data and the final responsibility to submit for publication.

## Results

We identified a family of European descent with an unusual clustering of childhood ALL at St. Jude Children's Research Hospital in the United States (Fig. 1): the mother (I-2) and 2 of her 3 daughters (II-2 and II-3) developed ALL at the ages of 9, 3, and 2, respectively. All 3 ALL cases were of B-lineage, although with different leukemia molecular subtypes. Mild thrombocytopenia was noted for I-2 and II-3 (appendix p 3), II-2 was diagnosed with Turner syndrome and mild intellectual disability, and II-3 was diagnosed with a learning disability (Table 1). The family history did not reveal other hematologic malignancies within the extended kindred.

We hypothesized that predisposition to ALL in this family was driven by a rare but highly penetrant germline genetic variation. Whole exome sequencing of all 5 family members was performed and variants were first prioritized on the basis of bioinformatic function annotation (e.g., damaging effects, gene function). We postulated that the causal variant should follow an autosomal dominant mode of inheritance and thus would be recurrent in ALL cases but absent in the unaffected father. We identified a total of 9 nonsilent variants that followed this pattern of segregation with ALL and were also rare in non-ALL controls (minor allele frequency [MAF] <0.01%), of which *ETV6* was the most likely candidate because of its known involvement in ALL pathogenesis (appendix p 2, 7). This nonsense variant in *ETV6* (c.1075C>T, p.R359X) was predicted to create a stop codon within the ETS domain and result in a truncated protein without DNA-binding function and was shared by all 3 ALL cases. Interestingly, it was also present in the healthy daughter (II-1), suggesting incomplete penetrance. However, it is formally possible that she was still at risk of ALL given her young age of 11. All carriers in the index family were heterozygous for this *ETV6* variant. Consistent with recent reports of recurring germline *ETV6* mutations associated with familial thrombocytopenia and overrepresentation of hematologic malignancies,^29-31^ this p.R359X variant in *ETV6* is likely to be responsible for the ALL predisposition in this family.

**Figure 2:**
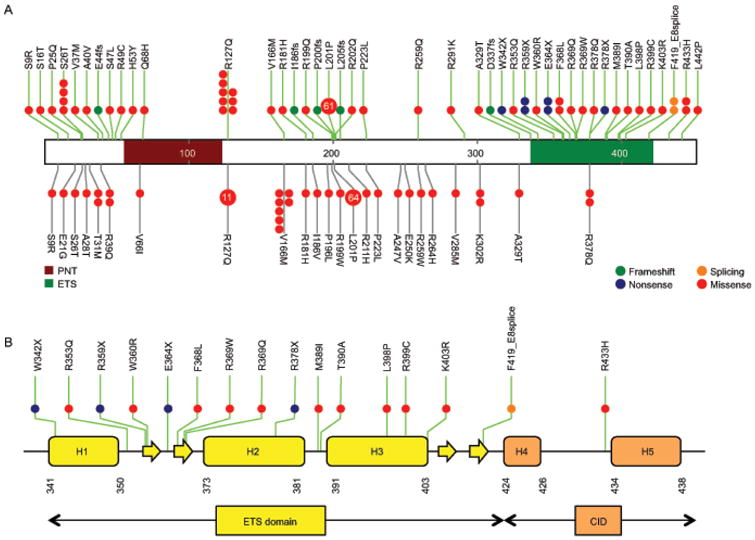
*ETV6* variants identified by targeted sequencing in 4,405 unselected ALL cases **(A)** *ETV6* was sequenced using Illumina HiSeq platform following capture-based enrichment of this genomic region in 4,405 unselected ALL cases. Variants in non-ALL controls were based on publicly available data from the NHLBI Exome Sequencing Project cohort (N=6,503). Exnoic variants are classified as frameshift, nonsense, missense, and splicing (green, blue, red, or orange solid circles, respectively) for ALL cases (green vertical lines) and non-ALL controls (gray vertical lines). Functional domains are indicated by color based on Pfam annotation. Each circle represents a unique individual carrying the indicated variant (heterozygous or homozygous) except variants recurring in more than 10 individuals for which the number in the circle indicates the exact frequency. **(B)** ALL-related *ETV6* variants are highly enriched in the ETS domain which consists of 3 helices (yellow boxes) and 4 β-sheets (yellow arrows). H3 is responsible for direct binding with DNA, which is negatively regulated by the CID domain (H4 and H5, orange boxes) at the C-terminal. Variant function is denoted by the color of each line.

To determine the overall prevalence of *ETV6*-related predisposition to childhood ALL, we performed targeted sequencing of *ETV6* in germline DNA from 4,405 children enrolled on St. Jude and the COG frontline clinical trials for newly diagnosed ALL. In this unselected cohort, we identified 43 exonic *ETV6* variants (Fig. 2A and appendix p 4-6). Of these, 12 *ETV6* variants were recurrent (e.g., MAF≥0.01%) in ancestry-matched non-ALL populations (the NHLBI GO Exome Sequencing Project cohort [ESP, N=6,503] and/or in the Exome Aggregation Consortium cohort [ExAC, N=60,706]) and thus less likely to be related to ALL risk. In contrast, 31 *ETV6* variants were observed only in children with ALL or were exceedingly rare in non-ALL populations (MAF<0.01%) and thus were considered as “ALL-related”. All but 5 ALL-related *ETV6* variants were singletons. In total, 35 (0.79%) of 4,405 children in this cohort had rare *ETV6* variants that were potentially related to ALL predisposition, including 4 nonsense, 21 missense, 1 splice, and 5 frameshift variants.

Fifteen of the 31 ALL-related *ETV6* variants (48.4%) were clustered within the ETS DNA-binding domain (Fig. 2B), while only 6 were expected if mutations were randomly distributed in *ETV6* (P=3.4×10^-4^). These 15 included all 4 nonsense variants (p.W342X, p.R359X, p.E364X, and p.R378X), 10 missense variants (p.R353Q, p.W360R, p.F368L, p.R369W, p.R369Q, p.M389I, p.T390A, p.L398P, p.R399C, and p.K403R), and 1 splice variant (F419_E8splice). Using the combined annotation dependent depletion (CADD) algorithm, we predicted 18 of the ALL-related *ETV6* variants to be highly deleterious (CADD phred-like score> 20). The p.D337fs variant had the highest CADD phred-like score (99), followed by 4 nonsense variants (score 40 for p.W342X, p. R359X, and p.E364X, and 39 for p.R378X). Compared with CADD phred-like scores of exonic *ETV6* variants observed in the non-ALL controls (i.e., the ExAC cohort), the ALL-related *ETV6* variants were significantly more likely to be damaging (mean CADD phred-like score of 25.6 vs 15.2; p<0.0001. appendix p 9). Interestingly, of the 18 most deleterious *ETV6* variants, 10 resided in the ETS domain but none were located in the helix that directly interacts with target DNA.^49^ Instead, 7 of the 10 variants in the ETS domain were between the first and second helices (Fig. 2B).

We next analyzed the relationship between germline risk variants in *ETV6* and clinical features of ALL, in a subset of 2,021 cases that were comprehensively characterized for clinical features and representative of the US childhood ALL population across risk and demographic groups (Table 2). Children with ALL-related *ETV6* variants were significantly older at the time of diagnosis than those without these variants (10.2 years [IQR 5.3-13.8] vs 4.7 years [IQR 3.0-8.7], P=0.017). The hyperdiploid leukemia karyotype was strikingly overrepresented in ALL cases harboring germline risk variants in *ETV6* compared to the wildtype group (9 [64.3%] of 14 cases vs 538 [26.8%] of 2,007 cases), P=0.0050). In contrast, the frequency of somatic *ETV6*-*RUNX1* fusion was only 7.1% (1 of 14) in cases with germline *ETV6* risk variants, compared to 22.7% (455 or 2,007) in cases with wildtype *ETV6* in the host genome. Of note, there was also a trend towards overrepresentation of females in carriers of ALL-related *ETV6* variants (71.4% [10 of 14] vs 45.7% [918 of 2,007], respectively), although it did not reach statistical significance. These germline *ETV6* risk variants were not associated with genetic ancestry, early treatment response (minimal residual disease at the end of remission induction therapy, P=0.29), or the risk of relapse (P=0.36).

## Discussion

In this study, we systematically identified germline variants in the *ETV6* gene that are associated with increased risk to childhood ALL, and comprehensively described clinical characteristics unique in children with ALL carrying these risk alleles. Inherited genetic predisposition to ALL refers to an increased likelihood of developing this cancer that is attributable to germline (constitutional) genetic variations. The contribution of germline genetic variants to ALL risk is now well established, particularly through genome-wide association studies.^10-16^ However, the susceptibility conferred by these common variants is usually modest (1.5-2-time increase in ALL risk for each copy of the variant allele).^50^ In contrast, a smattering of congenital genetic disorders have been linked to ALL predisposition (e.g., *PAX5*,^21^ *SH2B3*^22^ and *TP53*^23^ germline mutations), resulting in a dramatic increase in disease risk. However, these risk variants often have incomplete penetrance and the accompanying non-malignant symptoms can be mild.^17^ Therefore, it is conceivable that a familial cause will not be routinely suspected and it is challenging to accurately determine the extent to which sporadic ALL is attributable to rare germline predisposition variants. This is exemplified by the wide spectrum of ALL risk alleles identified in *ETV6* in our current study: close to 1% of unselected sporadic ALL cases carry likely damaging and potentially highly penetrant germline risk variants in a single gene *ETV6*. Our findings challenge the current paradigm that ALL is primarily driven by somatic genetic alterations and imply that the inherited genetic predisposition to this cancer may be much greater than previously believed. Thus, these data from us and others^29-31^ provide a strong rationale for including family history examination as part of the standard approach to the diagnosis and work up of pediatric ALL. For example, the family history should determine the presence of leukemia, the types of leukemia, the ages at leukemia diagnosis, and other hematological abnormalities (e.g., antecedent thrombocytopenia). However, future studies to comprehensively characterize the clinical features and natural course of ALL with inherited predisposition are needed to define clear guidelines for clinical actions appropriate for at-risk patients.

The unusual clustering of ALL-related *ETV6* variants within the critical ETS domain suggests that the loss or alteration of DNA-binding function of *ETV6* may be critical to the promotion of leukemogenesis. Experimental characterization of other *ETV6* variants in the ETS domain indicates that they may function in a dominate-negative manner in that the loss-of-function variant protein still oligomerizes with wildtype ETV6, thus altering subcellular localization and dramatically abrogating transcriptional repression^29, 30^. We hypothesize that this may be also true for ALL-related *ETV6* variants identified in the current study (especially those in the ETS domain), and thus predict a dominant transmission of disease phenotype in individuals carrying these risk alleles. In contrast, the function of *ETV6* variants outside of the ETS domain is much less understood and it is possible that they alter gene function in an additive manner with dosage effects on ALL predisposition. Interestingly, the ETS domain is also truncated in the *ETV6*-*RUNX1* fusion protein as a result of the somatic t(12;21) translocation and the remaining copy of wildtype *ETV6* is often subsequently deleted in overt leukemia^51^. In fact, we analyzed 15 ALL cases carrying germline *ETV6* risk variants with materials available and identified 2 with acquired copy number loss of *ETV6* in ALL blasts (not shown). Although common in childhood ALL in general,^28, 52^ the *ETV6*-*RUNX1* fusion is underrepresented in children who carry the *ETV6* germline risk alleles (Table 2), and this mutual exclusivity might imply possible overlap in the molecular mechanisms by which somatic and germline *ETV6* variations influence ALL leukemogenesis. For example, *ETV6*-*RUNX1* fusion arises *in utero* prenatally as one of the first acquired genomic abnormalities during the development of ALL^53^, and thus the constitutional germline *ETV6* variation may also play a role at the very early stage of leukemogenesis. Because both types of genetic changes negatively impact ETV6 function, it is conceivable that they render vulnerability to subsequent oncogenic events affecting common signaling pathways.

However, it should be noted that a critical function of *ETV6-RUNX1* is to disrupt RUNX1-mediated transcription regulation^54^ which may be distinct from the effects of *ETV*6 germline variants. *ETV6* is indispensable for the survival of adult hematopoietic stem cells but with little effect on their progeny.^24, 55^ Selective inactivation of *ETV6* in lineage committed progenitor cells showed profound defects in terminally differentiated megakaryocytes (reduction of platelet counts),^55^ consistent with the thrombocytopenia observed in patients with loss-of-function *ETV6* germline variants (Table 1).^29-31^ In contrast, B cell development is unaffected by lineage-specific *ETV6* deletion in mice^55^ and a distinct mechanism may exist to explain the increased risk in B-ALL conferred by germline defects in *ETV6*.

The possible incomplete penetrance of *ETV6* variants in the index family strongly argues that secondary mutations (most likely somatic) are needed for overt leukemogenesis, consistent with the relatively late disease onset in individuals who carry the *ETV6* risk variants (Table 2). Paradoxically, the frequency of hyperdiploid ALL (usually more common in young children) was significantly greater in this group despite their older age. One can hypothesize that somatic lesions characteristic of the hyperdiploid karyotype (e.g., mutations in the *RAS* pathway) may act synergistically with *ETV6* germline variants during leukemogenesis, which should be tested experimentally in future studies. Also of interest, there were 8 additional germline variants identified from whole exome seq of the index family (*CEP95*, *CEP250*, *GUSB*, *SNTG1*, *AGBL1*, *RAB7A*, *RSPRY1*, and *PRR23C*, appendix p 2) that also co-segregated with ALL and were rare (MAF<0.01%) in non-ALL controls. While there is no obvious link of these genes with leukemogenesis, their functions have been sparsely characterized and it remains unknown whether they also contributed to ALL risk in this family (especially for the few variants predicted to be deleterious by CADD).

In conclusion, we identify germline mutations in the gene encoding the critical hematopoietic transcription factor *ETV6* that co-segregate with ALL and thrombocytopenia in a rare leukemia-prone kindred. This observation, as well as similar recent findings^29-31^ suggests the presence of a novel genetic syndrome characterized by a predisposition to ALL and thrombocytopenia with a common underlying genetic cause (i.e., *ETV6* germline variants). We also comprehensively analyzed a large cohort of unselected pediatric ALL patients and identified that close to 1% of patients harbor potential predisposing germline *ETV6* variants in *ETV6*. Future longitudinal family studies are needed to better define the penetrance, age specific leukemia incidence and clinical features characterizing this syndrome. Similarly, experimental characterization is warranted to define the exact function of the potential ALL-related *ETV6* variants and to elucidate the molecular pathways by which they influence leukemia formation. These clinical and mechanistic studies are absolutely critical for the development of recommendations for clinical interventions in the future for individuals harboring ALL-related *ETV6* variants.

## Supplementary Material

1

## Figures and Tables

**Table 1:** Clinical features of individuals in the studied pedigree with familial ALL

Individual	*ETV6* genotype	ALL status	Age at ALL diagnosis (years)	Leukemia karyotype	Cytopenias	Additional Features
I-1	Wild Type	None	NA	NA	None	None
I-2	p.R359X	pre-B ALL	9	56,XX,+X,+4,+6,+10,+11,+14, +del(17)(p11.2),+18,+21,+21	Thrombocytopenia	None
II-1	p.R359X	None*	NA*	NA	None	None
II-2	p.R359X	pre-B ALL	3	46,X,i(X)(q10)c	None	Turner syndrome
II-3	p.R359X	pre-B ALL	2	46,X,i(X)(q10),del(6)(q13q21)	Thrombocytopenia	Learning disability

Abbreviations: ALL, acute lymphoblastic leukemia

* This individual was 11 and did not have leukemia at the time when this manuscript was written

**Table 2:** Clinical characteristics of ALL cases with or without risk variants in *ETV6*^a^

	COG9904/5 /6 (N=1,445)		SJ T13A/T13 B/T15 (N=576)		Combined (N=2,021)		P value
	Carriers of *ETV6* variants ALL-related		Carriers of *ETV6* variants ALL-related		Carriers of *TV6* variants ALL-related *E*		
	Yes N=8	No N=1,437	Yes N=6	No N=570	Yes N=14	No N=2,007	
**Age at diagnosis, ears**	12.0	4.5	7.1	5.8	10.2	4.7	0.017*
(median [range, IQR])	(4.6-15.4, 7.0-13.4)	(1.0-20.6, 3.0-7.7)	(2.0-15.4, 3.2-13.0)	(0.1-18.8, 3.3-10.9)	(2.0-15.4, 5.3-13.8)	(0.1-20.6, 3.0-8.7)	

**Leukocyte count 10^9^/l**							
<50	7 (87.5%)	1,229 (85.5%)	6 (100%)	419 (73.5%)	13 (92.9%)	1,648 (82.1%)	0.49**
≥50	1 (12.5%)	205 (14.3%)	0 (0%)	151 (26.5%)	1 (7.1%)	356 (17.7%)	
Unknown	0 (0%)	3 (0.2%)	0 (0%)	0 (0%)	0 (0%)	3 (0.1%)	

**DNA index**^b^							
≥1.16	4 (50%)	404 (28.1%)	5 (83.3%)	134 (23.5%)	9 (64.3%)	538 (26.8%)	0.0050**
<1.16	4 (50%)	989 (68.8%)	1 (16.7%)	433 (76.0%)	5 (35.7%)	1,422 (70.9%)	
Unknown	0 (0%)	44 (3.1%)	0 (0%)	3 (0.5%)	0 (0%)	47 (2.3%)	

**Gender**							
Male	2 (25%)	775 (53.9%)	2 (33.3%)	311 (54.6%)	4 (28.6%)	1,086 (54.1%)	0.063**
Female	6 (75%)	659 (45.9%)	4 (66.7%)	259 (45.4%)	10 (71.4%)	918 (45.7%)	
Unknown	0 (0%)	3 (0.2%)	0 (0%)	0 (0%)	0 (0%)	3 (0.1%)	

**Tumor translocation**							
*ETV6-RUNX1*	1 (12.5%)	354 (24.6%)	0 (0%)	101 (17.7%)	1 (7.1%)	455 (22.7%)	0.21**
*E2A-PBX1*	1 (12.5%)	68 (4.7%)	0 (0%)	31 (5.4%)	1 (7.1%)	99 (4.9%)	0.51**
*MLL* rearrangements	0 (0%)	15 (1.0%)	0 (0%)	8 (1.4%)	0 (0%)	23 (1.2%)	1**
*BCR-ABL1*	0 (0%)	0 (0%)	0 (0%)	12 (2.1%)	0 (0%)	12 (0.6%)	1**
Others	6 (75%)	992 (69%)	6 (100%)	418 (73.3%)	12 (85.7%)	1,410 (70.3%)	
Unknown	0 (0%)	8 (0.6%)	0 (0%)	0 (0%)	0 (0%)	8 (0.4%)	

**Ancestry**^c^ (median %)							
European	99.0%	98.1%	99.0%	99.0%	99.0%	98.3%	0.79***
African	0.1%	0.3%	0.1%	0.2%	0.1%	0.3%	
Asian	0.5%	0.5%	0.5%	0.3%	0.5%	0.4%	
Native American	0.4%	0.5%	0.5%	0.4%	0.5%	0.5%	

P value was estimated using

* the Wilcoxon rank sum test,

** the Fisher's exact test and

*** the Firth logistic regression test.

a Data are presented as No. (%) of patients unless otherwise indicate.

b DNA index of ≥ 1.16 indicates cases with high hyperdiploidy (>50 chromosomes in ALL blasts).

c Genetic ancestry was estimated as European, African, Native American, and Asian using STRUCTURE, on the basis of genotypes at 30,000 randomly selected SNPs, with HapMap samples and indigenous Native American references as ancestral populations. Numbers represent median value for each patient group.
