# Supplementary material for: Germline Genetic Variation in ETV6 and Risk of Childhood Acute Lymphoblastic Leukemia: a Systematic Genetic Study
Source: Lancet Oncol. Author manuscript; Available in PMC 2016 Dec 1. (PMC4684709; doi:10.1016/S1470-2045(15)00369-1)
Supplement: 1 [file NIHMS737731-supplement-1.pdf]

Supplementary Appendix

Germline Genetic Variation in *ETV6* and Predisposition to Childhood Acute Lymphoblastic Leukemia

Takaya Moriyama, MD, Monika L. Metzger, MD, Gang Wu, PhD, Rina Nishii, Maoxiang Qian, PhD, Meenakshi Devidas, PhD, Wenjian Yang, PhD, Cheng Cheng, PhD, Xueyuan Cao, PhD, Emily Quinn MS, Susana Raimondi, PhD, Julie Gastier-Foster, PhD, Elizabeth Raetz, MD, Eric Larsen, MD, Paul L. Martin, MD, PhD, W. Paul Bowman, MD, Naomi Winick, MD, Yoshihiro Komada, MD, PhD, Shuoguo Wang, PhD, Michael Edmonson, BA, Heng Xu, PhD, Elaine Mardis, PhD, Robert Fulton, PhD, Ching-Hon Pui, MD, Charles Mullighan, MBBS, MD, William E. Evans, PharmD, Jinghui Zhang, PhD, Stephen P. Hunger, MD, Mary V. Relling, PharmD, Kim E. Nichols, MD, Mignon L. Loh, MD, and Jun J. Yang, PhD

CONTENTS **PAGE**

SECTION 1: Tables

Table S1: Candidate germline variants identified by whole exome seq of the index family 2

Table S2: Complete blood count for carriers of the *ETV6* p.R359X variant in the familial ALL pedigree 3

Table S3: Germline *ETV6* variants identified in childhood ALL 4-6

SECTION 2: Figures

Figure S1: Flowchart of filtering and prioritization process to identify candidate variants related to ALL predisposition in the index family 7

Figure S2: Definition for *ETV6* variant types 8

Figure S3: CADD phred-like score of germline *ETV6* exonic variants 9

***Table S1*: Candidate germline variants identified by whole exome seq of the index family**

Gene	RefSeq	Affected subjects	Function	Nucleotide change	Protein change	dbSNP	COSMIC	ClinVar	SIFT	Polyphen	CADD phred-like score
ETV6	NM_001987	I-2, II-1, II-2, II-3	Nonsense	c.1075C>T	p.R359X	NA	NA	NA	NA	NA	40
CEP95	NM_138363	I-2, II-1, II-2, II-3	Splice	c.590G>A	p.A198_E7splice	NA	NA	NA	NA	NA	15.9
CEP250	NM_007186	I-2, II-1, II-2, II-3	Missense	c.6878A>G	p.N2293S	NA	NA	NA	Deleterious	Damaging	11.2
GUSB	NM_000181	I-2, II-1, II-2, II-3	Missense	c.896A>C	p.Y299S	NA	NA	NA	Deleterious	Damaging	14.5
SNTG1	NM_018967	I-2, II-1, II-2, II-3	Missense	c.383C>T	p.A128V	NA	NA	NA	Deleterious	Damaging	25.2
AGBL1	NM_152336	I-2, II-2, II-3	Missense	c.2510C>A	p.A834D	NA	NA	NA	Deleterious	Damaging	18.8
RAB7A	NM_004637	I-2, II-2, II-3	Missense	c.167T>C	p.L56P	NA	NA	NA	Tolerated	Benign	17.4
RSPRY1	NM_133368	I-2, II-2, II-3	Splice	c.1162-1G>A	p.E388_E11splice	NA	NA	NA	NA	NA	28.2
PRR23C	NM_001134657	I-2, II-2, II-3	Missense	c.350A>G	p.D117G	rs188589355	NA	NA	Tolerated	Benign	11.7

***Table S2*: Complete blood count for carriers of the ETV6 p.R359X variant in the familial ALL pedigree**

Individual	*ETV6* genotype	WBC ($x10^{12}$/l)	RBC ($x10^{12}$/l)	Hgb (g/dl)	HCT (g/dl)	PLT ($x10^{9}$/l)	MCV (fl)	MCH (pg)	MCHC (g/dl)	RDW (%)	MPV (fl)	ANC ($x10^{9}$/l)
I-2	p.R359X	5.7	3.54	12.0	33.5	122	94.7	33.9	35.8	12.8	7.8	3,700
II-1	p.R359X	8.3	4.02	12.7	37.4	181	92.9	31.5	33.9	13.0	8.2	2,600
II-2	p.R359X	6.9	4.34	13.1	39.4	217	90.8	30.3	33.4	13.8	7.9	3,800
II-3	p.R359X	8.4	4.17	12.7	37.2	129	89.3	30.5	34.2	13.3	7.4	5,000

Abbreviations: WBC, White Blood Cells; RBC, Red Blood Cells; Hgb, Hemoglobin; HCT, Hematocrit; PLT, Platelet; MCV, Mean Corpuscular Volume; MCH, Mean Corpuscular Hemoglobin; MCHC, Mean Corpuscular Hemoglobin Concentration; RDW, Red Cell Distribution Width; MPV, Mean Platelet Volume; ANC, Absolute Neutrophil Count.

Table S3: Germline *ETV6* variants identified in childhood ALL[a,b]

	Non-ALL cohorts		ALL cohort (N=4,405)								
Amino acid change	Variant frequency in ESP (%)	Variant frequency in ExAc (%)	Ancestry of variant carriers	Variant frequency (%)	dbSNP	COSMIC	ClinVar	SIFT	Polyphen	CADD phred-like score	Classification
p.S16T	NA	NA	European (N=1)	0.0114	NA	NA	NA	Tolerated	Probably damaging	18.49	ALL-related variant
p.P25Q	NA	NA	Others (N=1)	0.0114	NA	NA	NA	Tolerated	Probably damaging	15.67	ALL-related variant
p.E44fs	NA	NA	European (N=1)	0.0114	NA	NA	NA	NA	NA	22.5	ALL-related variant
p.H53Y	NA	NA	European (N=1)	0.0114	NA	NA	NA	Tolerated	Damaging	26.7	ALL-related variant
p.I186fs	NA	NA	Hispanics (N=1)	0.0114	NA	NA	NA	NA	NA	11.49	ALL-related variant
p.P200fs	NA	NA	European (N=1)	0.0114	NA	NA	NA	NA	NA	21.2	ALL-related variant
p.L205fs	NA	NA	European (N=1)	0.0114	NA	NA	NA	NA	NA	21.9	ALL-related variant
p.D337fs	NA	NA	Africans (N=1)	0.0114	NA	NA	NA	NA	NA	99	ALL-related variant
p.W342X	NA	NA	Hispanics (N=1)	0.0114	NA	NA	NA	Tolerated	NA	40	ALL-related variant
p.R359X	NA	NA	European (N=2)	0.0227	NA	NA	NA	Tolerated	NA	40	ALL-related variant
p.W360R	NA	NA	Others (N=1)	0.0114	NA	NA	NA	Deleterious	Damaging	24.6	ALL-related variant
p.E364X	NA	NA	European (N=2)	0.0227	NA	NA	NA	Deleterious	NA	40	ALL-related variant
p.F368L	NA	NA	European (N=1)<br>Others (N=1)	0.0227	NA	NA	NA	Deleterious	Damaging	34	ALL-related variant
p.R369Q	NA	NA	Others (N=1)	0.0114	rs724159946	NA	Pathogenic	Deleterious	Damaging	36	ALL-related variant
p.R369W	NA	NA	European (N=1)	0.0114	NA	NA	NA	Deleterious	Damaging	26.1	ALL-related variant
p.R378X	NA	NA	European (N=1)	0.0114	NA	NA	NA	Tolerated	NA	39	ALL-related variant
p.M389I	NA	NA	European (N=1)	0.0114	NA	NA	NA	Deleterious	Damaging	15.03	ALL-related variant
p.T390A	NA	NA	European (N=1)	0.0114	NA	NA	NA	Deleterious	Damaging	14.33	ALL-related variant
p.L398P	NA	NA	European (N=1)	0.0114	NA	NA	NA	NA	Damaging	13.2	ALL-related variant
p.R399C	NA	NA	European (N=1)	0.0114	rs724159945	NA	Pathogenic	Deleterious	Damaging	12.13	ALL-related variant
p.K403R	NA	NA	European (N=1)	0.0114	NA	NA	NA	Tolerated	Damaging	14.74	ALL-related variant
p.F419_ E8 splice	NA	NA	European (N=2)	0.0227	NA	NA	NA	NA	NA	31	ALL-related variant
p.R433H	NA	NA	European (N=2)	0.0227	NA	NA	NA	Tolerated	Probably damaging	25	ALL-related variant
p.L442P	NA	NA	Hispanics (N=1)	0.0114	NA	NA	NA	Tolerated	Damaging	16.68	ALL-related variant

	Non-ALL cohorts		ALL cohort (N=4,405)								
**Amino acid change**	**Variant frequency in ESP (%)**	**Variant frequency in ExAc (%)**	**Ancestry of variant carriers**	**Variant frequency (%)**	**dbSNP**	**COSMIC**	**ClinVar**	**SIFT**	**Polyphen**	**CADD phred-like score**	**Classification**
p.S9R	0.0077 (total)<br>0.0227 (African)	0.000829 (total)<br>0.00981 (African)	African (N=1)	0.0114	rs372541278	NA	NA	Tolerated	Benign	10.74	Common variant
p.S26T	0.0077 (total)<br>0.0116 (European)	0.0206 (total)<br>0.0328 (European)<br>0.00864 (Hispanic)	European (N=1)<br>Hispanic (N=2)<br>Unknown (N=1)	0.0454	rs150858928	NA	NA	Tolerated	Benign	11.69	Common variant
p.V37M	NA	0.00165 (total)<br>0.00273 (European)	European (N=1)	0.0114	NA	NA	NA	Tolerated	Probably damaging	19.37	ALL-related variant
p.A40V	NA	0.0355 (total)<br>0.248 (South Asian)<br>0.00273 (European)	Unknown (N=1)	0.0114	NA	NA	NA	Tolerated	Benign	18.5	Common variant
p.S47L	NA	0.00166 (total)<br>0.00274 (European)	Hispanic (N=1)	0.0114	NA	NA	NA	Tolerated	Benign	16.65	ALL-related variant
p.R49C	NA	0.00166 (total)<br>0.00864 (Hispanic)<br>0.0116 (East Asian)	Hispanic (N=1)	0.0114	NA	NA	NA	Deleterious	Damaging	25.8	ALL-related variant
p.Q68H	NA	0.000825 (total)<br>0.00865 (Hispanic)	Others (N=1)	0.0114	rs202004830	NA	NA	Tolerated	Benign	8.44	ALL-related variant
p.R127Q	0.0846 (total)<br>0.093 (European)<br>0.0681 (African)	0.0527 (total)<br>0.0804 (European)<br>0.0480 (African)	European (N=4)<br>Unknown (N=4)	0.0908	rs140357643	NA	NA	Tolerated	Probably damaging	17.75	Common variant
p.V166M	0.0538 (total)<br>0.159 (African)	0.0663 (total)<br>0.694 (East Asian)<br>0.00138 (European)<br>0.0771 (African)<br>0.112 (Others)<br>0.0600 (South Asian)	East Asian (N=1)	0.0114	rs142603082	NA	NA	Tolerated	Benign	9.115	Common variant
p.R181H	0.0077 (total)<br>0.0116 (European)	0.0107 (total)<br>0.0116 (East Asia)<br>0.0150 (European)<br>0.00606 (South Asia)	European (N=1)	0.0114	rs150089916	NA	NA	Tolerated	Damaging	23.4	Common variant
p.R199Q	NA	0.00412 (total)<br>0.00273 (European)<br>0.0182 (South Asia)	Hispanics (N=1)	0.0114	NA	NA	NA	Tolerated	Damaging	22.1	ALL-related variant
p.L201P	0.492 (total)<br>0.663 (European)<br>0.159 (African)	0.498 (total)<br>0.135 (African)<br>0.719 (European)<br>0.363 (Hispanic)<br>0.109 (South Asia)<br>0.330 (Others)	European (N=41)<br>African (N=2)<br>Hispanic (N=6)<br>Others (N=5)<br>Unknown (N=7)	0.692	rs145477191	NA	NA	Tolerated	Probably damaging	12.11	Common variant

	Non-ALL cohorts		ALL cohort (N=4,405)								
**Amino acid change**	**Variant frequency in ESP (%)**	**Variant frequency in ExAc (%)**	**Ancestry of variant carriers**	**Variant frequency (%)**	**dbSNP**	**COSMIC**	**ClinVar**	**SIFT**	**Polyphen**	**CADD phred-like score**	**Classification**
p.R202Q	NA	0.0198 (total)<br>0.00818 (European)<br>0.00864 (Hispanic)<br>0.0969 (South Asia)<br>0.110 (Others)	Others (N=1)	0.0114	rs200351280	NA	NA	Tolerated	Benign	12.13	Common variant
p.P223L	0.0077 (total)<br>0.0116 (European)	0.00247 (total)<br>0.00409 (European)	European (N=1)	0.0114	rs373315577	NA	NA	Tolerated	Benign	5.062	Common variant
p.R259Q	NA	0.00165 (total)<br>0.00273 (European)	Others (N=1)	0.0114	NA	Present	NA	Tolerated	Benign	17.37	ALL-related variant
p.R291K	NA	0.0124 (total)<br>0.175 (East Asian)	Others (N=1)	0.0114	rs72550787	NA	NA	Tolerated	Benign	15.85	Common variant
p.A329T	0.0077 (total)<br>0.0116 (European)	0.0163 (total)<br>0.0102 (African)<br>0.0238 (European)<br>0.00872 (Hispanic)	Africans (N=1)	0.0114	rs184240587	NA	NA	Tolerated	Probably damaging	22.2	Common variant
p.R353Q	NA	0.00329 (total)<br>0.00545 (European)	European (N=1)	0.0114	NA	NA	NA	Tolerated	Damaging	36	ALL-related variant
p.R378Q	0.0154 (total)<br>0.0233 (European)	0.0140 (total)<br>0.0219 (European)<br>0.110 (Others)	East Asian (N=1)	0.0114	rs146280653	NA	NA	Tolerated	Damaging	28.9	Common variant

[a]The majority of ALL-related *ETV6* variants are not described in dbSNP, COSMIC, or Clinvar.
[b]If a variant is not found in the databases, no numbers are listed in this table.

***Figure S1*: Flowchart of filtering and prioritization process to identify candidate variants related to ALL predisposition in the index family*.**

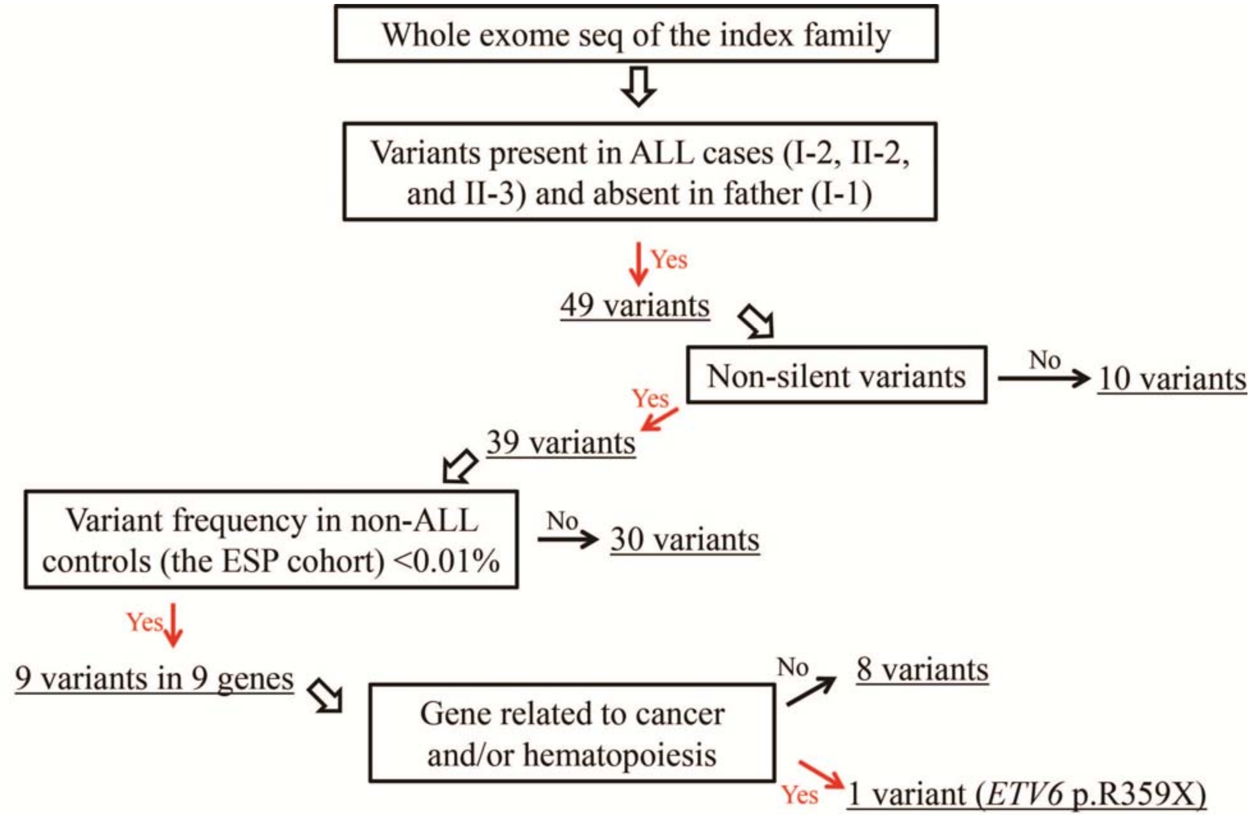

*Red arrows indicate the path that leads to the p.R359X *ETV6* variant.

***Figure S2*: Definition for *ETV6* variant types**

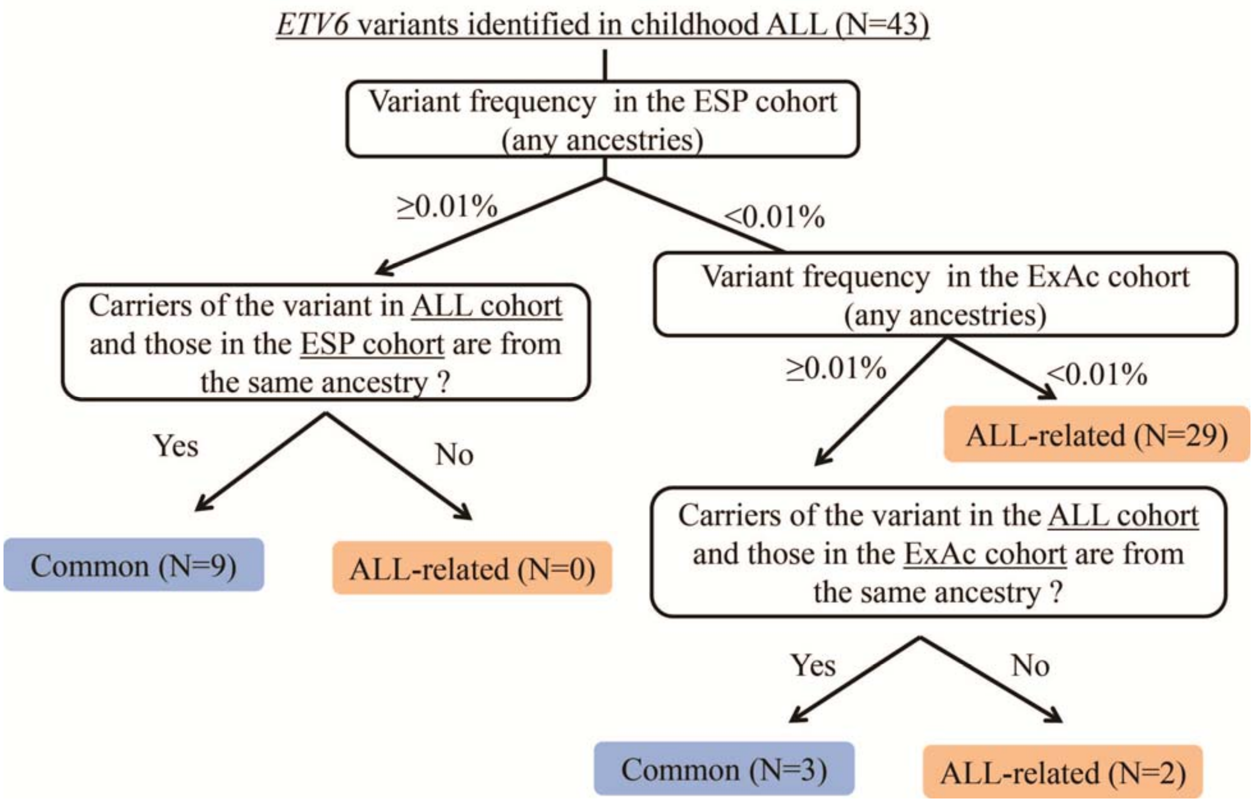

Figure S3*: CADD phred-like score of germline *ETV6* exonic variants

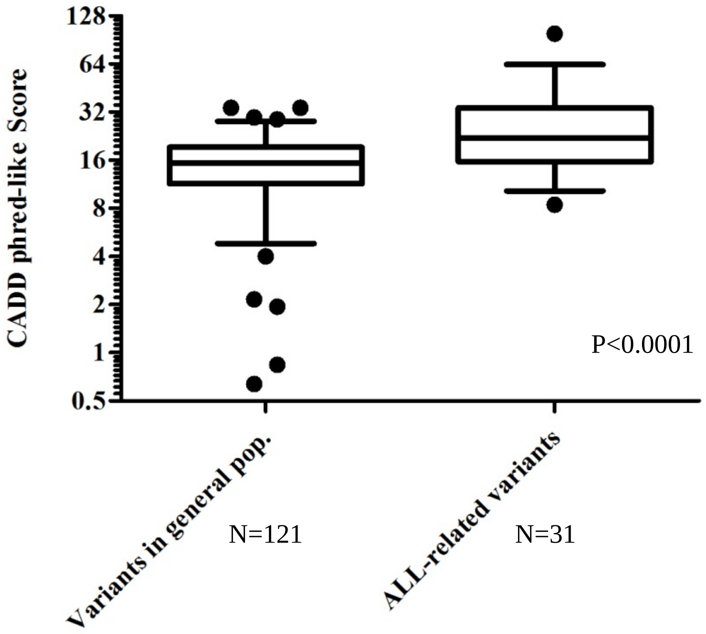

*Variants in general populations are based on data from the Broad Institute ExAc cohort (118 missense and 3 frameshift). The definition of ALL-related *ETV6* variants was described in Methods.
